# Radiomics analysis in medical imaging research

**DOI:** 10.1002/jmrs.662

**Published:** 2023-02-10

**Authors:** Mahmoud Elmahdy, Ronnie Sebro

**Affiliations:** ^1^ Department of Radiology Mayo Clinic Jacksonville Florida USA; ^2^ Center for Augmented Intelligence Mayo Clinic Jacksonville Florida USA; ^3^ Department of Orthopedic Surgery Mayo Clinic Jacksonville Florida USA; ^4^ Department of Biostatistics Centre for Quantitative Health Sciences Jacksonville Florida USA

## Abstract

This article discusses the current research in the field of radiomics in medical imaging with emphasis on its role in fighting coronavirus disease 2019 (COVID‐19). This article covers the building of radiomic models in a simple straightforward manner, while discussing radiomic models potential to help us face this pandemic.

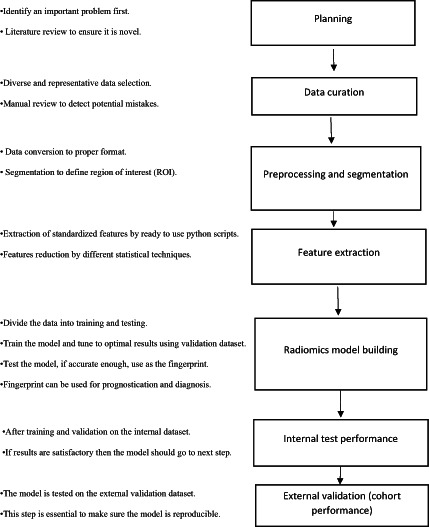

Radiomics are quantifiable features that are extracted from medical imaging data. These features are based on imaging features including pixel intensity, pixel arrangement, pixel colour and texture.^1^, ^2^ Radiomics in essence allows the conversion of an image into quantitative variables that can be processed by a software. Many features can be extracted from medical images using radiomics. While the human eye may utilise some of these features to classify images, subtle changes in radiomic features are often below the perceptible threshold of the human eye and can be undetectable to humans.^2^ Radiologists' analysis of imaging studies is currently considered the gold standard for diagnosis and prognosis,^3^ however, recent studies have shown that radiomic features extracted can further assist radiologists with diagnostic and prognostic healthcare challenges.^1^ This additional information extracted from medical images promises to change radiology and usher in a new era of personalised medicine.^4^, ^5^

Radiomic analysis requires seven key steps as shown in Figure 1: (1) planning (identification of a clinical question, study design, and literature review), (2) data curation, (3) data pre‐processing and image segmentation, (4) radiomic feature extraction, (5) radiomic model building and performance assessments (6) internal hold‐out test and (7) external validation datasets.^3^

**Figure 1:**
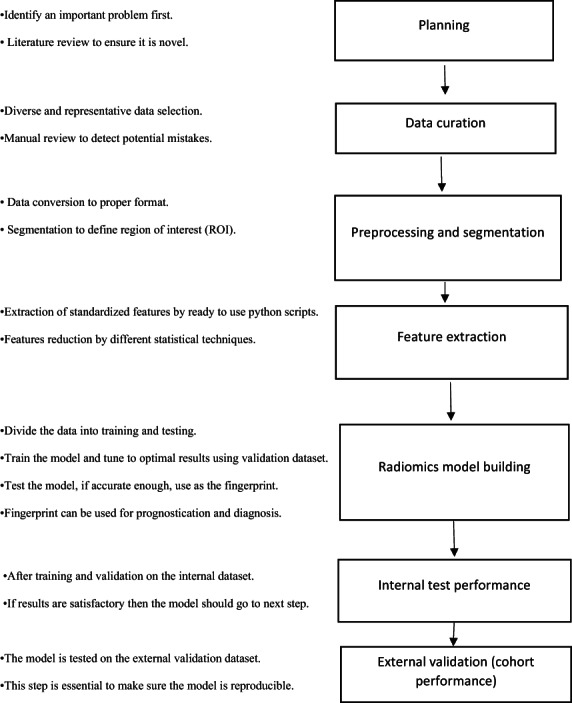
Steps of radiomics analysis in medical imaging research.

## Planning

The most important aspect of radiomic model building is identifying an important clinical problem that needs to be solved. The study must have the correct study design and sufficient statistical power to appropriately evaluate radiomic models.^3^ A detailed literature review should be performed to ensure that the authors are aware of all previous work on the topic.

## Data Curation

Data should be diverse and representative of the patient population and be obtained from a variety of different sources (scanners/machines) at different sites/centres. This helps create models that are able to be generalised to other similar patient populations and imaging devices.^6^ Data should, if possible, be manually reviewed to ensure no artefacts, incorrect data or incorrect labelling.

## Data Pre‐Processing and Image Segmentation

Image processing normally involves many steps after data download including data conversion, image pre‐processing, segmentation, interpolation and intensity discretisation.

Imaging data is normally stored in Digital Imaging and Communications in Medicine (DICOM) format.^7^ Single static DICOM images can be converted into Portable Network Graphics (PNG) or Joint Photographic Experts Group (JPEG) format, undergo image intensity normalisation and resizing, to ensure that images are the same size.

Image pre‐processing is an important step to enhance reliability of the radiomic features that are extracted. This should be done through a standardised approach.^3^

Segmentation is where regions of interest (ROI) are identified. Segmentation may be performed manually by a radiologist or other imaging expert, or in a semi‐automatic, or completely automated fashion.^8^ ROIs are used to define the specific region in which radiomic features are calculated. For segmentation analysis, DICOM images can be imported into several programs including 3D Slicer, ImageJ and ITK‐SNAP.^9^, ^10^, ^11^ The segmentation masks can be saved in several different formats, including The Neuroimaging Informatics Technology Initiative (NIfTI) format.^7^

Interpolation or estimation of the number of data points within the range of the data set which is important to reduce directional biases and ensure isotropic voxel spacing. This is necessary for most radiomic texture features to become rotationally invariant and to increase reproducibility of these radiomic features between different datasets.^2^

Intensity discretisation is where a discrete value or number is assigned to pixel intensities. for example grey‐level intensities, thereby scaling the pixel intensities into a given range.^12^, ^13^ Image intensity normalisation often follows discretisation, where the pixels may be normalised on a scale between 0 and 1. For segmentation analysis, the NIfTI files undergo image intensity normalisation and then freely available Python scripts can be used to extract radiomic features.

## Radiomic Feature Extraction

Ready to use and freely available Python scripts (e.g. PyRadiomics) can extract standardised radiomic features approved by the Image Biomarker Standardisation Initiative (IBSI).

Radiomic feature extraction from images are categorised as intensity based‐statistical, intensity histogram‐based, intensity‐volume histogram‐based, morphological features, local intensity and texture matrix‐based features according to IBSI.^12^ These categorisations are based on how the features are calculated.

Ideally, radiomic features should be reproducible, which means that the same radiomic feature values will be obtained if the data set were re‐analysed the same way.^2^, ^6^ There are several thousand radiomic features that can be extracted from a single image. Feature reduction selects only the important radiomic features to be used in the final model and is therefore an important aspect of model building.^14^

One of the challenges encountered doing radiomic analysis is that any image can generate several thousand radiomic features. It is difficult to know a priori which of these radiomic features are most important for model building. Several different radiomic feature reduction techniques including least absolute shrinkage and selection operator (LASSO), principal components analysis (PCA), linear discriminant analysis (LDA), support vector machines (SVM) and correlation coefficient analysis have been used.^14^, ^15^, ^16^

## Radiomic Model Building and Validation

Ideally there should be two independent data sets – an internal data set and external validation data set.^3^ The internal data set should be randomly split into training, validation and internal test data sets. The split should be done at the patient level, which ensures that the internal test and the training and validation data sets are independent.^3^

If radiomic features are reproducible, they can be used to create a radiomics model. This radiomics model could be used for patient categorisation, prognostication, response to treatment and other clinically important endpoints.^17^ One major challenge with radiomic analysis is that the model performance is not reproducible and often fails to replicate in external data sets.^18^, ^19^ Data availability, transparency and utilising IBSI‐approved radiomic features may help radiomics research to be more reproducible and help advance the field. Pre‐defined radiomic features may be used in radiomic model building. One benefit of using pre‐defined radiomic features is that the model created is more understandable to the user.^20^ Another option is to use deep learning techniques, which can be used to extract even more radiomic features and be used to create a better performing model.^21^ However, one downside of using deep learning AI is that the models created are ‘blackbox’, which means we do not know how the models are working. This leads to models with limited interpretability and understandability.^17^ Models are trained by optimising pre‐determined loss functions.

Model validation occurs after model training. Tuning is done as part of the validation step and is essential because poor tuning of the parameters may result in the model overfitting the data. Tuning is done through hyperparameter optimisation and cross‐validation. At the end of the tuning step, the best set of the hyperparameters are identified for the creation of the optimal tuned model.^22^

## Internal Test

The optimal tuned model performance should be assessed in the internal test and external validation datasets. The area under the curve (AUC) and the F1‐measure should be reported especially in cases of class imbalance. If the results are satisfactory the radiomic model should undergo external validation to assess generalisability.

## External Validation

The optimal tuned model performance should also be evaluated in an independent external validation data set. This step is essential to ensure the radiomics model generalises to data sets other than the internal data set and that the model is not overfit to the training data set.

Radiomics analysis has been used in several fields in medicine utilising imaging such as cardiology, ophthalmology and radiology. More recently radiomic analysis has been utilised in the field of thoracic radiology. Clinically important problems that have been addressed using radiomic analysis include (1) predicting patient prognosis in patients with lung cancers (2) predicting patients with lung cancer's response to therapy and which type of therapy is optimal (3) predicting whether lung tumours have certain molecular subtypes or genetic mutations, (4) predicting whether a pulmonary nodule is benign or malignant and requires biopsy.^8^, ^23^, ^24^, ^25^

Recent studies have also shown that radiomic analysis of computed tomography (CT) scans of the chest can differentiate patients with coronavirus disease 2019 (COVID‐19) from patients with other pneumonia of different aetiologies.^26^, ^27^, ^28^ Radiomic analysis has been shown to predict (a) whether a patient who is positive for COVID‐19 needs to be hospitalised (b) to predict whether a patient who is positive COVID‐19 would need the intensive care unit (ICU) and/or ventilators^29^, ^30^, ^31^ (c) how long a patient with COVID‐19 is expected to be hospitalised (d) future mortality risk from COVID‐19 and (e) predicting whether patients with COVID‐19 go on to develop long COVID‐19.^30^, ^31^, ^32^, ^33^, ^34^ These data can be used to identify patients at increased risk for clinical deterioration from COVID‐19 and could help appropriately allocate COVID‐19 resources ahead of time including determining ICU beds/ventilators, CT scan intervals and clinicians/healthcare providers needed for patients.^35^

The American College of Radiology (ACR) does not advocate use of chest CT for the initial evaluation of patients with COVID‐19.^36^ In the early stages of the COVID‐19 pandemic, prior to the advent of rapid antigen tests, COVID‐19 PCR testing took several hours.^37^ Chest radiographs (CXR) in conjunction with clinical symptoms were used to triage patients and to determine whether patients could quarantine at home or whether patients needed to be kept in the hospital for medical observation/treatment while PCR tests were pending. Radiomic analysis of CXR has been used to (a) predict whether a patient with pneumonia has COVID‐19 or some other pneumonia (b) predict whether patients would need hospitalisation or ventilation/ICU.^7^, ^38^, ^39^, ^40^, ^41^

Nakashima et al.^41^ used chest radiographs (CXR) to predict ‘danger of death’ in patients with COVID‐19 with 90.9% and 95.6% sensitivity and specificity respectively, emphasising the role of radiomics in facing COVID‐19 pandemic. This study should have an internal test data set to assess the model performance in the internal test data set and would ideally be followed by more studies and papers with larger data sets and external validation (testing) data sets from different hospitals to make sure the models are reproducible and generalisable.^3^ Radiomic models that predict worse outcomes for a patient with COVID‐19 in a low resource area where there is limited access to CT and limited availability of hospital beds may help hospitals rapidly identify these patients and transfer these patients to other hospitals so that the patients' outcomes improve.^38^

In summary, radiomic analysis of imaging data is a rapidly growing field that can be used to advance medicine by extracting more data from images than is readily available to a human observer or radiologist. Radiomic analysis has been used to assist patient management and treatment decisions for patients with COVID‐19.^30^ In the future, we anticipate that radiomics research will increase and eventually be incorporated as the standard in clinical radiology practice.

## Conflict of Interest

The author declares no conflict of interest.
